# AGouTI–Flexible Annotation of Genomic and Transcriptomic Intervals

**DOI:** 10.1371/journal.pcbi.1011527

**Published:** 2023-10-18

**Authors:** Jan G. Kosiński, Marek Żywicki

**Affiliations:** Department of Computational Biology, Institute of Molecular Biology and Biotechnology, Faculty of Biology, Adam Mickiewicz University in Poznań, Poznań, Poland; University of Washington, UNITED STATES

## Abstract

The recent development of high-throughput workflows in genomics and transcriptomics revealed that efficient annotation of such results is essential for researchers to draw conclusions from obtained results. Although some tools are available, their functionality is limited. Here, we present AGouTI–a universal tool for flexible annotation of any genomic or transcriptomic coordinates using known genomic features deposited in different publicly available databases in the form of GTF or GFF files. In contrast to currently available tools, AGouTI is designed to provide a flexible selection of genomic features overlapping or adjacent to annotated intervals and can be used on custom column-based text files obtained from different data analysis pipelines. Although providing many unique options, AGouTI is straightforward in installation and usage, enabling effortless integration into existing data analysis workflows.

## Introduction

In recent years, the increased adaptation of high-throughput sequencing techniques to solve complex research questions has revolutionized the field of molecular biology. This was possible by inference of numerous experimental protocols, followed by estimation of specific data analysis pipelines. In most data analysis scenarios, the sequences derived from NGS experiments are at some point aligned to the reference genome or transcriptome to infer their location. The comparison of obtained coordinates with the location of known genomic features allows to obtain comprehensive information about regions of interest in the context of genes, regulatory regions, UTRs, and CDSs. Several tools for so-called genomic arithmetics have been developed to enable efficient comparison of genomic locations, with BEDOPS [1] and BEDTools [2] as the most widely used software suites for genomic interval analysis and annotation. These tools allow for manipulating and analyzing sets of genomic intervals and performing operations such as intersection, union, and difference. The primary purpose of these tools is to provide a set of command-line utilities for genomic arithmetic, which allows users to quickly and efficiently manipulate genomic intervals and perform complex analyses.

BEDTools is a comprehensive suite of tools for genomic interval analysis. It is widely used for manipulating and analyzing genomic intervals stored in the BED, GFF, or VCF format. Some commands most commonly used for annotating intervals include *intersect* and *closest*. The *intersect* command allows users to identify genomic intervals that overlap between two datasets, while the *closest* command enables the identification of the closest intervals from one dataset to another.

BEDOPS is another widely used suite of tools for genomic interval analysis. Similarly, as BEDTools, BEDOPS is designed to provide a fast and scalable set of tools for set operations on genomic intervals. The suite includes commands such as *bedmap* and *closest-features*. The *closest-features* command, for example, enables users to identify the closest features from one dataset to another. Thus, it can be helpful in identifying regulatory elements or genes closest to intervals of interest.

Several other genomic interval analysis and annotation tools have been developed in recent years, including annotatePeaks.pl from Homer [3] or AnnotateGenomicRegions [4]. However, while all mentioned tools are well adapted for annotating genomic coordinates, none can work with transcriptomic data in transcript-based coordinates. Such datasets are usually obtained from analysis of the transcriptome-oriented NGS experiments, such as identification of alternative splicing events, protein or miRNA binding sites, and RNA editing sites. One of the most prominent examples illustrating the requirement for annotation of the transcript-based coordinates comes from recently developed protocols for high throughput transcriptome-wide RNA structure probing with protocols such as structure-seq [5] or SHAPE-MaP [6]. Due to the transcript-specific nature of those experiments, all the data analysis is performed using transcript sequences as a reference. Thus, all the obtained results, including the structural signal distribution and modeled secondary structures, are described in transcript-based coordinates. A similar situation can be observed in the case of the identification of miRNA binding sites. Since their presence and activity could differ among alternative transcript isoforms, all the results from experiments, like degradome sequencing or computational prediction of target sites, are defined in reference to positions in specific transcripts rather than within the genome. Moreover, due to the lack of standardized tools for arithmetics of transcriptome-based coordinates, most transcript-oriented data analysis pipelines provide results in custom text tables instead of standardized formats such as BED, GTF/GFF, or VCF.

To fill this gap, we have developed *AGouTI*–a tool for flexible Annotation of Genomic and Transcriptomic Intervals. *AGouTI* was designed to annotate any genomic or transcriptomic regions using user-selected features from genome annotations stored in GTF or GFF files. It allows researchers to understand better the transcriptome and how it relates to the genome. With this information, researchers can gain more insights into gene expression, alternative splicing, and other vital biological processes at the transcriptomic level. *AGouTI’s* ability to annotate transcriptomic intervals provides a valuable tool that will enable further exploration of the transcriptome’s intricacies and enhance the understanding of the biological processes underpinning it.

## Design and implementation

### AGouTI workflow

**Fig 1:**
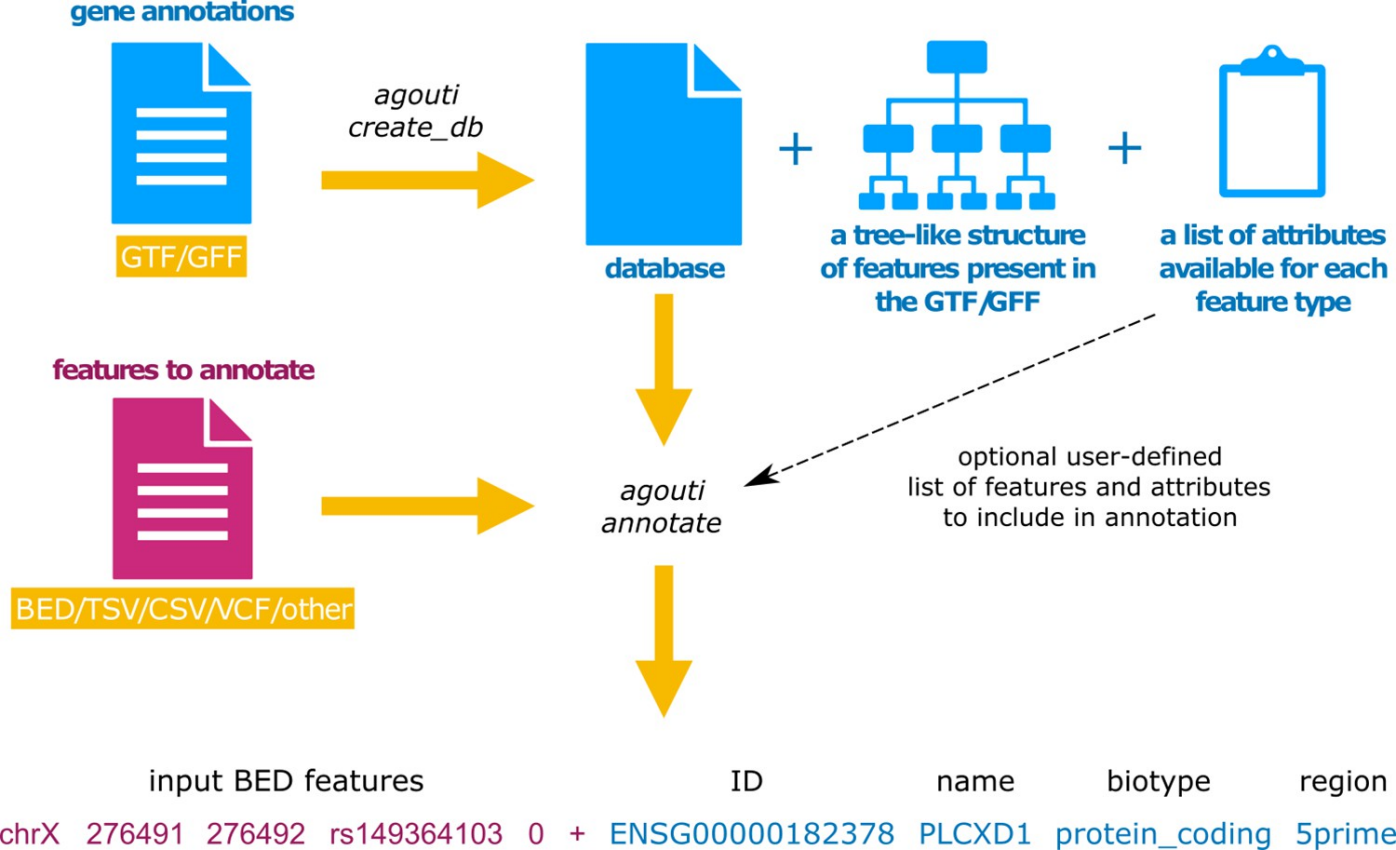
Schematic representation of the annotation process with *AGouTI*.

**Fig 2:**
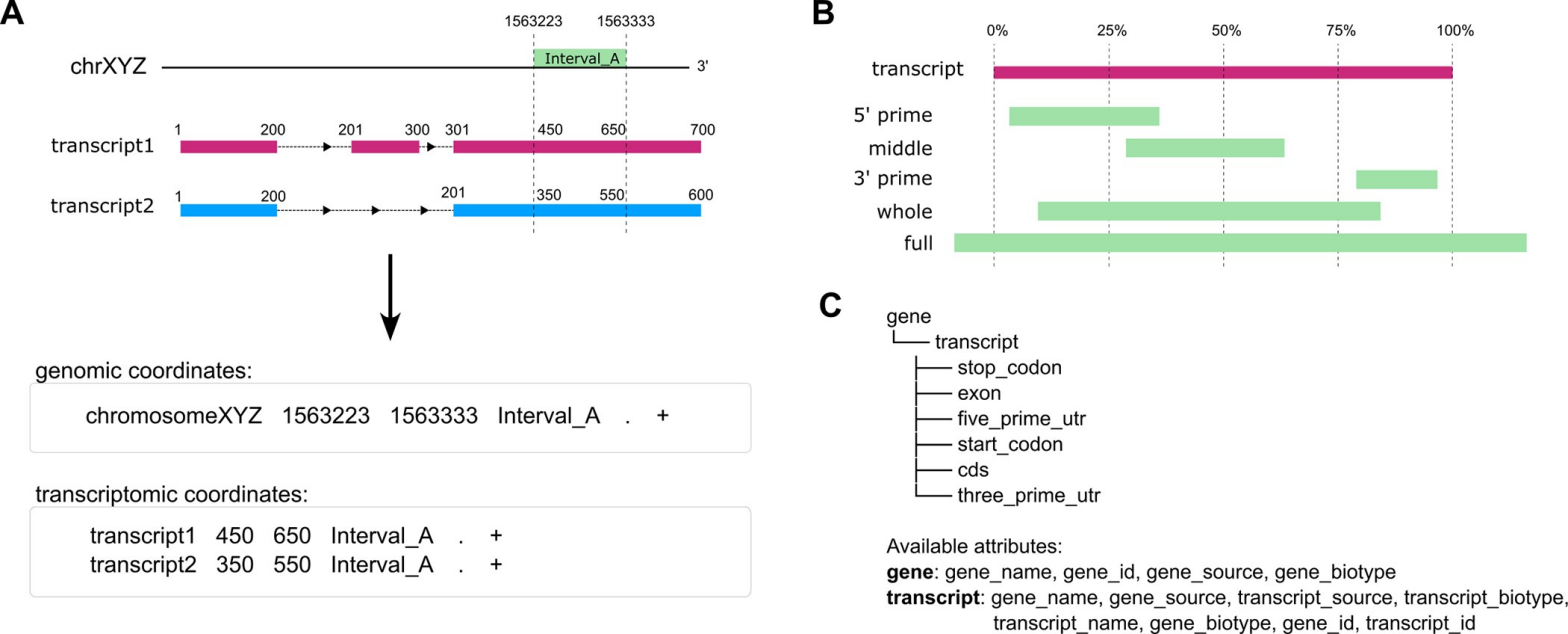
Overview of key features of *AGouTI*. (A) The interdependence between genomic and transcriptomic coordinates. (B) The de novo assignment of the region to the intervals overlapping with transcript features. (C) The GTF feature hierarchy as provided by *agouti create_db* after inspection of the GTF file along with example lists of attributes available for selection during annotation for gene and transcript features.

*AGouTI* has been designed to provide an efficient, flexible, and universal framework for the annotation of the genomic and transcriptomic intervals generated by various tools and workflows as a result of the high-throughput analysis of biological data. To achieve high annotation efficiency, the *AGouTI* workflow has been divided into two steps–the creation of the annotation database, followed by the annotation itself, requiring to run two simple commands: *agouti create_db* and the *agouti annotate* (Fig 1). In the first step, the SQLite database is created based on genome annotations provided by the user in a GTF or GFF file (*agouti create_db* command). The employment of a relational database enables fast and memory-efficient selection of genomic features and their attributes during annotation. To provide a comprehensive GTF/GFF format combability, the *gffutils* library [7] is used for parsing input files and feature extraction. The features’ names and attributes are automatically converted to lowercase to enable case-insensitive use of the database in the following steps of the *AGouTI* pipeline. The SQLite database is initially created in the memory to speed up the process. However, users can initiate the process to be fulfilled directly on a hard drive, substantially reducing the required memory by the pipeline’s running time cost. Next, the database is inspected to provide and visualize a hierarchical list of features and corresponding attributes fetched from the GTF/GFF file, which will be available for annotation (Fig 2C). Finally, the database is written to the hard drive, so it needs to be created only once for each GTF/GFF file and can be reused in multiple projects, significantly reducing the analysis time.

Once the annotation database has been created, the *agouti annotate* command can be run to annotate user-provided intervals with overlapping features stored in the SQL database. *AGouTI* supports multiple formats of the user-provided input file, containing intervals for annotation, including standard formats (e.g., BED, VCF, and similar) and custom text tables. During the annotation, AGouTI automatically converts between transcript- or genome-based and 0- or 1-based coordinates when applicable. All overlapping features meeting the command-line specified selection and assignment criteria are added to each line of the interval file as additional columns. The results are displayed in a tabular and self-explanatory format on the standard output.

## Results

### AGouTI features

*AGouTI* provides several features unavailable in other annotation software (Table 1 and Fig 2). The significant advantage is the ability to annotate regions defined by genome- and transcript-based coordinates (Fig 2A). In genomic mode, the coordinates are expected to reflect genomic positions of the interval, with chromosome or scaffold ID as reference (1^st^ column of BED file). In such cases, gene, transcript, and all the child positional features (e.g., CDS, UTRs) defined in the GTF/GFF file are assigned to each interval by identification of the positional overlap. In contrast to other annotation tools, the intervals without gene/transcript overlap are also annotated. *AGouTI* marks them as “intergenic” and provides information about the closest gene up- and downstream. On the other hand, when running *AGouTI* in transcriptomic mode, the intervals are expected to reflect positions within the transcript, with transcript ID as a reference. In this case, the transcript and its paternal gene from the GTF/GFF file are assigned simply by mapping the transcript ID. The positions of all the child features concerning the transcript are recalculated to within-transcript coordinates and intersected with the positions of the annotated interval.

**Table 1:** Comparison of *AGouTI* features with other similar tools.

	AGouTI	BEDTools	BEDOPS	HOMER
Ability to assign intragenic regions (UTRs, CDS, etc.)	+	+	+	+
Annotation by genomic coordinates	+	+	+	+
Annotation of standard file formats (BED, VCF)	+	+	+	+
Direct support for GTF and GFF3 files	+	-	+	+
Annotation by transcriptomic coordinates	+	-	-	-
Flexible selection of attributes to include in annotation	+	-	-	-
Annotation of custom column-based text files	+	-	-	-
Inspection of the GTF/GFF3 file content and summary of the available attributes	+	-	-	-
Support for files in 0-based and 1-based coordinate systems	+	-	-	-

In both modes, *AGouTI* calculates the relative region of the interval within the overlapping transcript, providing information whether it is located within the 3’ or 5’ part, in the middle, spans over (almost) the whole, or contains the entire transcript (Fig 2B). The rules for region assignment rely on localization of start and stop coordinates within the appropriate quartiles of the transcript: for the 5’ part, the start must be within the first and end within the first or second quarter; for the 3’ part, the start must be within the third or fourth and end within fourth quarter; for the middle part, the start and end must be within the second and third quarter; for whole the start must be within the first and end within the fourth quarter, but the length of the annotated feature cannot exceed 90% of transcript length; for full the start must be within the first and end within the fourth quarter, but the length of the annotated feature exceed 90% of transcript length. Such rough assignment of the transcript region provides additional insight into the location of the annotated intervals, independent of the GTF-provided transcript regions, such as UTRs, which AGouTI also assigns.

The currently available tools usually perform annotation by simply joining the complete lines describing overlapping regions or by assigning a strictly defined set of information. In contrast, *AGouTI* has been designed to provide the highest possible flexibility and control over the selection of features and attributes that should be included in the annotation. First, during the creation of the database, the GFF/GTF file is inspected, and a comprehensive hierarchical list of all feature types and attribute classes available for them, which are incorporated into the annotation database, is presented to the user (Fig 2C). This allows the user to inspect and easily select which available information should be appended to annotated intervals. Next, performing the annotation, the user can choose to assign to intervals all available information (default) or select only those relevant to the data being annotated. For example, in the case of annotation of miRNA binding sites, the user can decide to limit the information to transcript ID, transcript name, and transcript region (5UTR, CDS, 3UTR). In contrast, when annotating SNPs, the information about overlaps with a stop codon, start codon, and exons can be relevant. By default, multiple transcripts overlapping a given interval are returned as individual lines, whereas all child features are appended as subsequent columns. It is, however, possible to decide whether the annotation should be performed on the transcript (default) or gene level. In the latter case, alternative gene transcripts are treated as child features and grouped into a single output line.

*AGouTI* can be easily adapted to annotate any column-based text files containing at least information about interval ID, reference (chromosome or transcript ID), and start and end positions. The columns containing specific information and custom column separator (default: tabulation) can be specified using appropriate program options. Users can also determine whether the coordinates provided for annotation are 0- or 1-based. Finally, *AGouTI* allows users to choose whether annotated intervals should be located entirely within the assigned features or partial overlaps should be included. Notably, the annotation is appended to each line of the input file as additional columns without alteration of the original format of the data (Table 2). Finally, *AGouTI* can provide the statistics of assigned features and attributes, e.g., counts of gene or transcript biotypes and CDS/UTR distribution, depending on a set of information selected for annotation (Fig B in S1 Text).

**Table 2:** Example output of the annotation of the transcriptomic intervals in custom format with *AGouTI*. The original columns of the input file (selected from psRNAtarget output for clarity) are marked with grey. The complete versions of the original and annotated tables are available in S1 Text.

feature_id	transcript_id	feature_start	feature_end	inhibition	multiplicity	annotated_gene_id	annotated_featutype	annotated_chromosome	annotated_transcript_start	annotated_transcript_end	cds	five_prime_utr	three_prime_utr	gene_biotype	transcript_biotype	feature_region
**3-bna-miR156a**	CDY69014	682	702	Cleavage	1	GSBRNA2T00082020001	transcript	LK038451	2498	3741	y	.	.	protein_coding	protein_coding	middle
**67-bna-miR156a**	CDY37684	463	483	Cleavage	1	GSBRNA2T00064576001	transcript	LK032406	72286	72999	.	.	y	protein_coding	protein_coding	3 prime
**68-bna-miR156a**	CDY57212	524	544	Cleavage	1	GSBRNA2T00020688001	transcript	LK033555	9849	10564	.	.	y	protein_coding	protein_coding	3 prime
**69-bna-miR156a**	CDX79544	593	613	Cleavage	1	GSBRNA2T00132295001	transcript	LK031818	616499	617340	.	.	y	protein_coding	protein_coding	3 prime
**70-bna-miR156a**	CDY17204	642	662	Cleavage	1	GSBRNA2T00095270001	transcript	LK032071	417632	418459	.	.	y	protein_coding	protein_coding	3 prime
**71-bna-miR156a**	CDY34028	862	882	Cleavage	1	GSBRNA2T00055896001	transcript	LK032326	266964	268151	y	.	.	protein_coding	protein_coding	3 prime
**72-bna-miR156b**	CDX80287	335	355	Cleavage	1	GSBRNA2T00133286001	transcript	LK031820	1212808	1218040	.	.	y	protein_coding	protein_coding	middle
**73-bna-miR156c**	CDX80287	335	355	Cleavage	1	GSBRNA2T00133286001	transcript	LK031820	1212808	1218040	.	.	y	protein_coding	protein_coding	middle

Although *AGouTI* contains many options allowing for extensive customization of the annotation process, it is straightforward to use. When using the default options (input file in BED format, selection of all available features and attributes), it requires specifying only the input files. An example use-case scenario is described in the S1 Text. The installation process is also user-friendly–the packages are available via Python Package Index (PyPI) and Anaconda Cloud. Additionally, we provide the Galaxy wrapper, which can be easily included in any Galaxy instance via Galaxy Toolshed.

### Annotation of miRNA binding sites with AGouTI

As the use-case of the *AGouTI* pipeline, we describe the annotation of miRNA target regions predicted in mRNA sequences of Brassica napus with the psRNATarget tool [8]. The psRNATarget provides results in TSV format with 14 columns describing the details of the predicted miRNA-target interactions. The information about target mRNAs is limited to mRNA accession numbers. To obtain additional annotation of miRNA target sites with *AGouTI*, one can use the psRNATarget file as the *custom* input and select the appropriate columns containing information about feature ID (miRNA_Acc., column 1), reference ID (Target_Acc., column 2), interval start (Target_start, column 7), and interval end (Target_end, column 8) (see Table A in S1 Text for psRNATarget file layout). As a result, *AGouTI* appends additional columns containing features of the miRNA target sites, including details about the gene and its genomic location, CDS/UTR location, relative position in a transcript, and gene/transcript biotype (Table 2). Thus, with a single invocation of AGouTI, we could provide comprehensive annotation of the predicted miRNA binding sites, enabling the interpretation of psRNATarget results in terms of potential regulatory impact. The detailed walk-through has been described in S1 Text.

### AGouTI performance

To get insight into the time and memory usage of *AGouTI*, we decided to use the above-described, most computationally demanding scenario employing the unique features of *AGouTI*–annotation of the transcriptomic intervals in custom format, including calculation of the intragenic location of the annotated intervals and generation of summary statistics. The detailed procedure, including the software invocation commands, has been provided in the use-case scenario described in S1 Text. To enable the performance measurements on large datasets, annotation features from *Brassica napus* GTF file and miRNA binding sites from *psRNATarget* have been multiplied to obtain up to 100,000 genes with their sub-features and up to 20,000 intervals, respectively. To simulate the real-life usage of *AGouTI*, benchmark runs were performed on a MacBook Air M1 laptop (2020) with 16GB RAM and calculated as an average of 10 independent command invocations. Measurements were taken using the *gtime* command.

**Fig 3:**
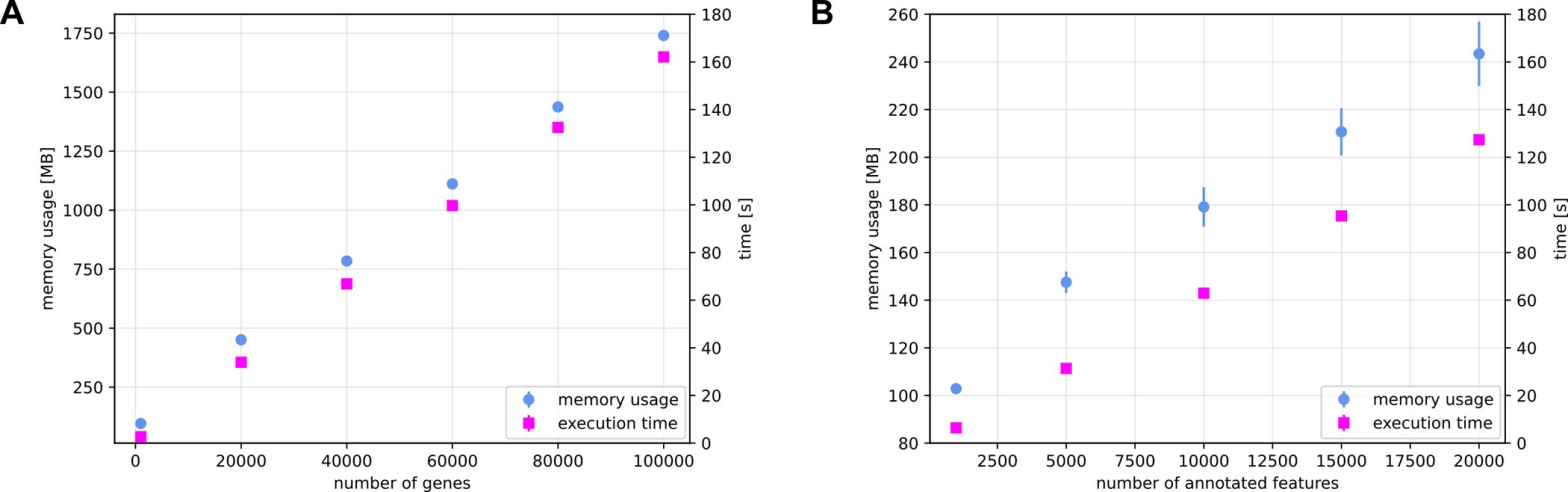
Benchmark results of *AGouTI* performed on the annotation of the miRNA binding sites in *Brassica napus*. (A) Dependence of memory usage and *agouti create_db* execution time on the number of genes included in processed GTF file. (B) Dependence of memory usage and *agouti annotate* execution time on the number of intervals provided for annotation. Error bars represent the standard deviation for ten repetitions.

The benchmark revealed that unique, computationally demanding features of AGouTI are accompanied by loss of computational complexity. However, in both steps–database build and annotation, the time and memory usage have linear complexity with a relatively small footprint, allowing efficient use of *AGouTI* on standard computer setups (Fig 3). The processing speed of the *agouti annotate* with the above compute-demanding combination of features was 160 intervals per second. The detailed benchmarking results have been presented in Fig 3.

### Discussion

AGouTI is the first tool that allows for the annotation of intervals based on both genome-based and transcript-based coordinates and provides flexibility in selecting features and attributes for annotation. The workflow involves two steps: creating an SQLite database based on user-provided genome annotations and using the database to identify features and attributes overlapping with genomic or transcriptomic intervals in column-based text files. The tool is user-friendly and offers high performance, as demonstrated by linear time and memory complexity for annotation and database building. Additionally, *AGouTI* includes unique features such as estimating intragenic regions and combining functionality from two different tools in the bedtools suite.

Compared with other existing tools, *AGouTI*, BEDTools, and BEDOPS are all software tools designed for interval analysis and annotation. However, several key differences between these tools make them unique and suitable for different applications. *AGouTI* is specifically designed to work with transcript-based coordinates, which allows for the annotation of transcriptomic intervals. *AGouTI* can annotate any genomic or transcriptomic region using user-selected features from genome annotations stored in GTF or GFF files. This makes *AGouTI* a valuable tool for researchers studying alternative splicing events, RNA editing, RNA binding, and other transcript-level phenomena. However, *AGouTI* does not offer the same level of comprehensive set operations as BEDTools or BEDOPS.

BEDTools and BEDOPS, on the other hand, are designed to perform set operations on genomic intervals and are widely used for genomic interval analysis and annotation. BEDTools and BEDOPS offer a comprehensive set of tools for manipulating and analyzing genomic intervals stored in BED, GFF, or VCF format. BEDTools and BEDOPS are both highly scalable and can be used to analyze large datasets with high efficiency. However, neither BEDTools nor BEDOPS is designed to work with transcript-based coordinates or custom file formats, which limits their utility for transcriptome analysis.

Overall, *AGouTI* provides researchers with a powerful tool to study transcriptomic data in a flexible and customizable way. With the ability to annotate any genomic or transcriptomic region using user-selected features from genome annotations stored in GTF or GFF files, *AGouTI* is an essential addition to the suite of high-throughput tools available to researchers.

## Availability and future directions

*AGouTI* is freely available under GNU General Public License v3.0 from GitHub (https://github.com/zywicki-lab/agouti), Python Package Index (https://pypi.org/project/AGouTI/), or Anaconda Cloud (https://anaconda.org/bioconda/agouti). We also provide a Galaxy wrapper available from the Galaxy Tool Shed (https://toolshed.g2.bx.psu.edu/view/janktoolshed/agouti/c204da8f836d). Data necessary for replicating the use-case example described in S1 Text is available from Zenodo (https://doi.org/10.5281/zenodo.7317210). We have also used Zenodo to assign a DOI to the GitHub repository (https://doi.org/10.5281/zenodo.7779492).

The future development of the *AGouTI* will focus on implementing additional features for increased functionality in the annotation of genomic intervals, which would broaden the area of *AGouTI* utilization. Moreover, we plan to implement the support for database creation using multiple GTF/GFF files, which would enable users to combine various sources of annotation.

## Supporting information

S1 Text Annotation of miRNA target sites with AGouTI. (PDF)
