# Supplementary material for: AGouTI–Flexible Annotation of Genomic and Transcriptomic Intervals
Source: PLoS Comput Biol. 2023 Oct 18;19(10):e1011527. doi: 10.1371/journal.pcbi.1011527 (PMC10621952; doi:10.1371/journal.pcbi.1011527)
Supplement: S1 Text — (PDF) [file pcbi.1011527.s001.pdf]

AGouTI - flexible Annotation of Genomic and Transcriptomic Intervals

Supplementary Information

Jan G. Kosiński, Marek Żywicki

Installation of the AGouTI

AGouTI is freely available on GitHub (https://github.com/zywicki-lab/agouti), from the Python Package Index (https://pypi.org/project/AGouTI/), and Anaconda Cloud (https://anaconda.org/bioconda/agouti). We also provide a Galaxy wrapper available from the Galaxy Tool Shed (https://toolshed.g2.bx.psu.edu/view/janktoolshed/agouti/c204da8f836d). The detailed installation instructtions, specific for the source of the software downlowad, are available under the above links.

Example use-case scenario

As the use-case of the AGouTI pipeline, we describe the annotation of miRNA target regions predicted in mRNA sequences of *Brassica napus* with the psRNATarget tool (https://www.zhaolab.org/psRNATarget/home) (Xinbin Dai et al. 2018, 2011, 2011). The output of the psRNATarget is a tab-separated text file containing information about predicted interaction sites, including their positions within the mRNA sequence. The input files necessary for completing the below-described workflow are available on Zenodo (DOI: 10.5281/zenodo.7317210; https://doi.org/10.5281/zenodo.7317210).

For prediction of the miRNA binding sites, the online version of *psRNATarget* has been used with default settings (as of 07/07/2022) on the input consisting of 92 *Brassica napus* miRNA sequences from the miRBase database (release 21, June 2014). *Brassica napus* transcriptome has been downloaded from the Ensembl Plants database (release 53) and restricted to the first 40 000 transcript sequences to limit the volume of the example dataset. The results have been downloaded and saved as the *psRNATarget_bnapus.tsv*. The structure of the file has been presented in Table A.

For annotation of the predicted miRNA binding sites in mRNA, we have downloaded the *Brassica napus* genome annotation in GTF format from the Ensembl Plants database (release 53; *Brassica_napus.AST_PRJEB5043_v1.53.gtf.gz*).

The first step of the AGouTI pipeline is to create a dedicated database based on the annotation file. The user can easily do this with the following command:

agouti create_db -f GTF -a Brassica_napus.AST_PRJEB5043_v1.53.gtf.gz -d bnapus.db

Average execution time: ~169s
Maximum memory usage: ~1796MB
(tested on MacBook Air M1 2020, with 16GB RAM, calculated as an average of 10 independent command invocations)

As a result, several files have been created, including the SQLite database (*bnapus.db*) and the file describing the structure, features, and attributes of the GTF file (*bnapus.db.database.structure.txt*; Fig A).

```
Attributes available for each feature type:

gene: gene_id, gene_biotype, gene_source, gene_name
transcript: gene_biotype, gene_source, gene_name, transcript_biotype, transcript_id, gene_id,
transcript_name, transcript_source
exon: gene_source, gene_biotype, gene_name, transcript_biotype, exon_id, transcript_id, gene_id,
exon_number, transcript_name, transcript_source
cds: gene_source, gene_biotype, gene_name, transcript_biotype, transcript_id, protein_id, gene_id,
exon_number, transcript_name, transcript_source
start_codon: gene_source, gene_biotype, gene_name, transcript_biotype, transcript_id, gene_id,
exon_number, transcript_name, transcript_source
stop_codon: gene_source, gene_biotype, gene_name, transcript_biotype, transcript_id, gene_id,
exon_number, transcript_name, transcript_source
five_prime_utr: gene_biotype, gene_source, gene_name, transcript_biotype, transcript_id, gene_id,
transcript_name, transcript_source
three_prime_utr: gene_biotype, gene_source, gene_name, transcript_biotype, transcript_id, gene_id,
transcript_name, transcript_source


gene
└── transcript
    ├── stop_codon
    ├── three_prime_utr
    ├── start_codon
    ├── five_prime_utr
    ├── exon
    └── cds
```

Fig A. The content of the *bnapus.db.database.structure.txt* file describing the input annotation file's structure and the attributes of each feature type available for selection during annotation.

Once the database is prepared, it is possible to run the annotation procedure. AGouTI requires each interval to have a unique ID. The *psRNATarget_bnapus.tsv* file doesn't have a column with such an ID, meaning it needs to be added before proceeding with the AGouTI pipeline. This can be done simply by adding in any spreadsheet software (e.g., Microsoft Excel) a column with unique ascending numbers or by running the following awk command:

awk 'NR>2{print(NR"-"$0)} NR <=2 {print}' psRNATarget_bnapus.tsv > psRNATarget_bnapus_with_ID.tsv

Tip! The value of 2 in the expressions "NR>2" and "NR <= 2" of the command corresponds to the number of header lines present in the file, which should be excluded from the process.

The annotation process can be run using:

agouti annotate -d bnapus.db -i psRNATarget_bnapus_with_ID.tsv -n 2 --custom 1,2,7,8 -r -t --statistics -p "\t" -a gene_biotype,transcript_biotype > agouti_miRNA-targets.tsv 2> agouti_statistics.txt

Average execution time: ~73s
Maximum memory usage: ~207MB
(12137 annotated features (lines), tested on MacBook Air M1 2020, with 16GB RAM, calculated as an average of 10 independent command invocations)

The annotated (input) *psRNATarget* result file (*psRNATarget_bnapus_with_ID.tsv*) is a TSV file with the custom column order (see Table A). Therefore, the user needs to specify (the --*custom* option) the column numbers containing feature id (1st column), transcript (reference sequence) id (2nd), start (7th), and end (8th) coordinates of the predicted interaction site. Column separator can be defined with the *-p* flag and the number of header

lines with -*n*. In our file, coordinates are described using a transcript-based coordinate system, thus, the option -*t* is also necessary. Although the intragenic regions defined in the GTF file, overlapping with annotated regions, are returned by default, the user can also decide to calculate the intragenic location of the entry from the input file by adding the -*r* flag. Possible values returned by this option are:

- **5 prime** - when the annotated interval starts within the first quarter of gene or transcript and ends in the first half
- **middle** - interval starts and ends within the second and third quarter, respectively
- **3 prime** - interval starts within the third quarter and ends in the last one
- **whole** - interval starts within the first quarter and ends within the last one. The annotated interval size **does not exceed** 90% of the transcript or gene length.
- **full** - interval starts within the first quarter and ends within the last one. The annotated interval size **exceeds** 90% of the transcript or gene length.
- **upstream** – interval is located upstream to the gene or transcript
- **downstream** – interval is located downstream to the gene or transcript.

Furthermore, the user can decide that the only valuable attributes to include in the annotation are '*gene_biotype*' and '*transcript_biotype*' by applying the -*a* flag. The --*statistics* option can be used to generate an additional summary displayed on the stderr by default (see *agouti_statistics.txt* and Fig B). The main output of the *agouti annotation* command is displayed on the stdout (see *agouti_miRNA-targets.ts*v and Table B).

```
####################
##STATISTICS
####################
# statistics for the 'cds' column: 'y' - 9963; '.' - 2173
# statistics for the 'five_prime_utr' column: '.' - 11285; 'y' - 851
# statistics for the 'three_prime_utr' column: '.' - 10703; 'y' - 1433
# statistics for the 'gene_biotype' column: 'protein_coding' - 12136
# statistics for the 'transcript_biotype' column: 'protein_coding' - 12136
# statistics for the 'feature_region' column: 'middle' - 5734; '3 prime' - 3449;
'5 prime' - 2948; 'other' - 5
```

Fig B. Statistics generated by the AGouTI pipeline based on the output file.

Supplementary references

1. Cunningham F, Allen JE, Allen J, Alvarez-Jarreta J, Amode MR, *et al.* (2018). psRNATarget: a plant small RNA target analysis server (2017 release). Nucleic Acids Research. doi: 10.1093/nar/gky316.
2. Xinbin Dai and Patrick X. Zhao (2011). psRNATarget: a plant small RNA target analysis server. Nucleic Acids Research 39(Web Server issue):W155-9. doi: 10.1093/nar/gkr319.
3. Xinbin Dai, Zhaohong Zhuang and Patrick X. Zhao (2011). Computational analysis of miRNA targets in plants: current status and challenges. Briefings in Bioinformatics 12(2):115-21. doi: 10.1093/bib/bbq065.

Table A. The sample rows of the *psRNATarget_bnapus.tsv* file as a schematic representation of the custom input file format.

#Please import the downloaded file into Microsoft Excel or other spreadsheet software													
**miRNA_Acc.**	Target_Acc.	Expectation	UPE$	miRNA_start	miRNA_end	Target_start	Target_end	miRNA_aligned_fragment	alignment	Target_aligned_fragment	Inhibition	Target_Desc.	Multiplicity
**bna-miR156a**	CDY69014	0.0	-1.0	1	21	682	702	UGACAGAAGAGAGUGAGCACA	:::::::::::::::::::::	UGUGCUCACUCUCUUCUGUCA	Cleavage		1
**bna-miR156d**	CDY69014	0.0	-1.0	1	20	683	702	UGACAGAAGAGAGUGAGCAC	::::::::::::::::::::	GUGCUCACUCUCUUCUGUCA	Cleavage		1
**bna-miR156e**	CDY69014	0.0	-1.0	1	20	683	702	UGACAGAAGAGAGUGAGCAC	::::::::::::::::::::	GUGCUCACUCUCUUCUGUCA	Cleavage		1
**bna-miR156f**	CDY69014	0.0	-1.0	1	20	683	702	UGACAGAAGAGAGUGAGCAC	::::::::::::::::::::	GUGCUCACUCUCUUCUGUCA	Cleavage		1
**bna-miR171f**	CDY56248	0.0	-1.0	1	21	774	794	UGAUUGAGCCGCGCCAAUAUC	:::::::::::::::::::::	GAUAUUGGCGCGGCUCAAUCA	Cleavage		1
**bna-miR171f**	CDX95633	0.0	-1.0	1	21	811	831	UGAUUGAGCCGCGCCAAUAUC	:::::::::::::::::::::	GAUAUUGGCGCGGCUCAAUCA	Cleavage		1
**bna-miR171f**	CDY67934	0.0	-1.0	1	21	799	819	UGAUUGAGCCGCGCCAAUAUC	:::::::::::::::::::::	GAUAUUGGCGCGGCUCAAUCA	Cleavage		1
**bna-miR171f**	CDY63062	0.0	-1.0	1	21	832	852	UGAUUGAGCCGCGCCAAUAUC	:::::::::::::::::::::	GAUAUUGGCGCGGCUCAAUCA	Cleavage		1

Tabke B. The sample rows of the *psRNATarget_bnapus.tsv* file annotated with the *AGouTI* pipeline.

#Please import the downloaded file into Microsoft Excel or other speadsheet software																									
**feature_id**	transcript_id	Expectation	UPE$	miRNA_start	miRNA_end	feature_start	feature_end	miRNA_aligned_fragment	alignment	Target_aligned_fragment	Inhibition	Target_Desc.	Multiplicity	annotated_gene_id	annotated_featutype	annotated_chromosome	annotated_transcript_start	annotated_transcript_end	cds	five_prime_utr	three_prime_utr	gene_biotype	transcript_biotype	feature_region
**3-bna-miR156a**	CDY69014	1	-1	1	21	682	702	UGACAGAAGAGAGUGAGCACA	::::::::::::::::::: ::	UGUGCUCACUCUCUUCUGUCA	Cleavage		1	GSBRNA2T00082020001	transcript	LK038451	2498	3741	y	.	.	protein_coding	protein_coding	middle
**67-bna-miR156a**	CDY37684	1	-1	1	21	463	483	UGACAGAAGAGAGUGAGCACA	: ::::..::::::::::::::	UUUGCUUGCUCUCUUCUGUCA	Cleavage		1	GSBRNA2T00064576001	transcript	LK032406	72286	72999	.	.	y	protein_coding	protein_coding	3 prime
**68-bna-miR156a**	CDY57212	1	-1	1	21	524	544	UGACAGAAGAGAGUGAGCACA	: ::::..::::::::::::::	UUUGCUUGCUCUCUUCUGUCA	Cleavage		1	GSBRNA2T00020688001	transcript	LK033555	9849	10564	.	.	y	protein_coding	protein_coding	3 prime
**69-bna-miR156a**	CDX79544	1	-1	1	21	593	613	UGACAGAAGAGAGUGAGCACA	: ::::..::::::::::::::	UUUGCUUGCUCUCUUCUGUCA	Cleavage		1	GSBRNA2T00132295001	transcript	LK031818	616499	617340	.	.	y	protein_coding	protein_coding	3 prime
**70-bna-miR156a**	CDY17204	1	-1	1	21	642	662	UGACAGAAGAGAGUGAGCACA	: ::::..::::::::::::::	UUUGCUUGCUCUCUUCUGUCA	Cleavage		1	GSBRNA2T00095270001	transcript	LK032071	417632	418459	.	.	y	protein_coding	protein_coding	3 prime
**71-bna-miR156a**	CDY34028	1	-1	1	21	862	882	UGACAGAAGAGAGUGAGCACA	: :::: ::::::::::::	UUUGCUCUCUCUCUUCUGUCA	Cleavage		1	GSBRNA2T00055896001	transcript	LK032326	266964	268151	y	.	.	protein_coding	protein_coding	3 prime
**72-bna-miR156b**	CDX80287	1	-1	1	21	335	355	UUGACAGAAGAUAGAGAGCAC	:::::::::::::::::	AAGCUCUCUAUCUUCUGUCAU	Cleavage		1	GSBRNA2T00133286001	transcript	LK031820	1212808	1218040	.	.	y	protein_coding	protein_coding	middle
**73-bna-miR156c**	CDX80287	1	-1	1	21	335	355	UUGACAGAAGAUAGAGAGCAC	:::::::::::::::::	AAGCUCUCUAUCUUCUGUCAU	Cleavage		1	GSBRNA2T00133286001	transcript	LK031820	1212808	1218040	.	.	y	protein_coding	protein_coding	middle
